# Childhood cancer risk in those with chromosomal and non-chromosomal congenital anomalies in Washington State: 1984-2013

**DOI:** 10.1371/journal.pone.0179006

**Published:** 2017-06-08

**Authors:** Marlena S. Norwood, Philip J. Lupo, Eric J. Chow, Michael E. Scheurer, Sharon E. Plon, Heather E. Danysh, Logan G. Spector, Susan E. Carozza, David R. Doody, Beth A. Mueller

**Affiliations:** 1 Public Health Sciences Division, Fred Hutchinson Cancer Research Center, Seattle, Washington, United States of America; 2 Department of Epidemiology, University of Washington, Seattle, Washington, United States of America; 3 Department of Pediatrics, Section of Hematology-Oncology, Baylor College of Medicine, Houston, Texas, United States of America; 4 Seattle Children’s Hospital, Seattle, Washington, United States of America; 5 Division of Epidemiology/Clinical Research, Department of Pediatrics, University of Minnesota, Minneapolis, Minnesota, United States of America; 6 College of Public Health and Human Sciences, Oregon State University, Corvallis, Oregon, United States of America; National Health Research Institutes, TAIWAN

## Abstract

**Background:**

The presence of a congenital anomaly is associated with increased childhood cancer risk, likely due to large effects of Down syndrome and chromosomal anomalies for leukemia. Less is known about associations with presence of non-chromosomal anomalies.

**Methods:**

Records of children diagnosed with cancer at <20 years of age during 1984–2013 in Washington State cancer registries were linked to their birth certificates (N = 4,105). A comparison group of children born in the same years was identified. Congenital anomalies were assessed from birth records and diagnosis codes in linked hospital discharge data. Logistic regression was used to estimate odds ratios (OR) and 95% confidence intervals (CI) for cancer, and for specific cancer types in relation to the presence of any anomaly and specific anomalies.

**Results:**

Having any congenital anomaly was associated with an increased risk of childhood cancer (OR: 1.46, 95% CI 1.28–1.65). Non-chromosomal anomalies were also associated with increased childhood cancer risk overall (OR: 1.35; 95% CI: 1.18–1.54), and with increased risk of several cancer types, including neuroblastoma, renal, hepatoblastoma, soft-tissue sarcoma, and germ cell tumors. Increasing number of non-chromosomal anomalies was associated with a stronger risk of childhood cancer (OR for 3+ anomalies: 3.11, 95% CI: 1.54–6.11). Although central nervous system (CNS) anomalies were associated with CNS tumors (OR: 6.05, 95% CI 2.75–13.27), there was no strong evidence of other non-chromosomal anomalies being specifically associated with cancer occurring in the same organ system or anatomic location.

**Conclusions:**

Non-chromosomal anomalies increased risk of several cancer types. Additionally, we found that increasing number of non-chromosomal anomalies was associated with a stronger risk of cancer. Pooling similar data from many regions would increase power to identify specific associations in order to inform molecular studies examining possible common developmental pathways in the etiologies of birth defects and cancer.

## Introduction

Congenital anomalies (i.e., birth defects) are one of the strongest and most consistent risk factors for childhood cancer. Birth defects are generally categorized as chromosomal or non-chromosomal anomalies.[1] The role of chromosomal anomalies on childhood cancer risk has been described. For example, children with Down syndrome (DS) have a 20-fold increased risk of acute lymphoblastic leukemia (ALL) compared to those without DS.[2, 3] Similarly, children with chromosome 13q14 deletion syndrome, characterized by dysmorphic facial features, have increased risk of retinoblastoma.[4] Four recent population-based registry linkage studies in the United States (U.S.)[2, 5–7] suggest that children with non-chromosomal anomalies may also be more likely to develop cancer compared to their unaffected contemporaries.

Evidence of shared biological pathways for congenital anomalies and cancer is limited, but possible mechanisms proposed include non-genetic exposures (e.g., environmental exposures) that lead to both conditions;[2] somatic mutations in developmental genes early in embryogenesis leading to tissue mosaicism;[8] or chromosomal microdeletions that include both developmental and cancer predisposition genes.[7] The biological underpinnings of these associations are likely to vary by specific birth defect and specific cancer type.

Few studies have evaluated possible associations of specific non-chromosomal anomalies with specific cancer types, largely due to the rarity of both childhood cancer and congenital anomalies. Relatively large study sizes can be conducted in different geographic regions using population-based linked health registry data allowing uniform measurement of both the congenital anomaly and cancer incidence. Such large linked databases provide rich opportunities to examine the associations of specific anomalies, particularly those that are not chromosomal in origin, with specific cancers. Using linked population-based birth-cancer-registry-hospital discharge data from Washington State in a case-control epidemiological study, we examined the relationships of congenital anomalies with childhood cancers, with a focus on major non-chromosomal anomalies.

## Materials and methods

### Subject identification

This project was conducted after appropriate Institutional Review Board approvals (expedited reviews with waivers of consent for data linkage to construct analysis files without names) were received from Washington State and the Fred Hutchinson Cancer Research Center. We linked records of all children <20 years old diagnosed with cancer in 1974–2014 as identified in the Washington State population-based cancer incidence registries to State birth records for the same years to identify children born in-state (N = 5,876). The cancer registries included the Surveillance, Epidemiology, and Endpoints (SEER) Program-affiliated Cancer Surveillance System of Western WA, and the Centers for Disease Control (CDC) National Program of Cancer Registries (NPCR)-affiliated Washington State Cancer Registry. Linkage of cancer registry and birth records databases was performed in a stepwise deterministic procedure based on identifiers contained within both resources including: child name, sex, and birth date; parental names and maternal birthdate; residential address at delivery and diagnosis; and race/ethnicity. Birth-hospital discharge records have been routinely linked since 1987 in Washington State and thus the ICD codes within the hospital discharge records and the birth record information were available from this linkage. Updated linkages of these cancer registry-birth records data have been conducted periodically during the past several decades, with birth records generally located for approximately 80% of cancer cases <15 at diagnosis (ranging from 66% - 85% of those 10–14, and <5 years old at diagnosis, respectively.) For each case, we randomly selected 10 control children without cancer during the study period from the remaining birth records, frequency matched on year of birth and sex (N = 58,462). Information about the presence of congenital anomalies began in the birth records in 1984, and thus our potential subjects included 4,590 cases and 45,653 controls born in 1984 or later. After excluding subjects with nonmalignant tumors (N = 480), and cervical cancers (N = 5) (due to their likely association with HPV infection), there were 4,105 cases for analyses.

### Congenital anomaly ascertainment

Washington birth certificates contain checkboxes indicating the presence of maternal and infant conditions, including congenital anomalies identified at delivery. Additionally, since 1987, Washington birth certificates have routinely been linked to hospital discharge records for the birth hospitalization of the infant; these were also used to identify congenital anomalies in case and control children, as birth certificate and hospital discharge records used in combination have been demonstrated to improve identification of several conditions,[9–11] and because birth certificate data enriched by hospital discharge information for identification of congenital anomalies has greater validity.[12] Washington State hospital discharge records include all hospital discharges in non-Federal facilities. For the study period, this state-wide system contains International Classification of Diseases-Clinical Modification, 9^th^ Revision (ICD-9) diagnosis codes for hospitalizations based on Medicare-Medicaid billing standards. During the study years, up to 25 diagnostic code fields were present for birth hospitalizations. We initially screened these for the presence of any congenital anomaly (ICD-9 740–759), and further refined by categorizing conditions as major or minor (S1 Table for ICD-9-CM codes).[13] This was further refined using a *Centers for Disease Control and Prevention/British Pediatric Association* (CDC/BPA)-modified code with greater detail. If the modified code for a congenital anomaly did not have a direct translation to ICD-9-CM, it was included as within the larger ICD-9-CM category. Anomaly types included: central nervous system (CNS); heart/circulatory; oral clefts; gastrointestinal; genital/urinary; chromosomal; musculoskeletal; integument/skin; and other congenital anomalies. Children with both a chromosomal (e.g., Down syndrome) and a non-chromosomal anomaly (e.g., oral cleft) were included in the “chromosomal anomaly” category.

#### Information available

Variables from the cancer registries included: ICD-O morphology and topography codes, stage, grade, histology, age at diagnosis, and diagnosis year. Cases were classified into groups and subtypes according to the International Classification of Childhood Cancer, 3^rd^ Edition,[14] and by age at diagnosis (<5, 5–9, 10–19 years). Additional information available from the birth records included demographic characteristics (e.g., parental age, race/ethnicity, education); maternal exposures and characteristics (e.g., prenatal smoking, marital status); and birth characteristics (birthweight, gestational length). Information about the type of medical insurance used for the child’s delivery or billed at hospital discharge was obtained from the birth certificate or from the hospital discharge record (categorized as private insurance vs. Medicaid/Medicare/Charity Care vs. private/other insurance) for use as a proxy indicator of socioeconomic status.

#### Analyses

Children were classified as indicated by birth certificate and/or hospital discharge data as having: any major congenital anomaly (with or without any congenital minor anomaly); minor congenital anomalies only; or no congenital anomaly. After initially evaluating the possible role of minor congenital anomalies for childhood cancer, the remainder of the analyses focused on major anomalies only. We evaluated the number of different types of congenital anomalies that a child had (e.g., CNS, gastrointestinal). If a child had two congenital anomalies within the same category, this was considered as having one type of anomaly. We then focused on non-chromosomal anomalies. We evaluated this association overall, and for cancer occurrence at different diagnosis age categories (<5, 5–9, 10–19 years) to be consistent with previous assessments.[5] Because congenital anomalies may be associated with infant birthweight or gestational age at delivery, which may also affect the risk of cancer occurrence, we conducted sub-analyses of our main exposures (any major anomaly, major non-chromosomal anomalies) restricted to children with normal birthweight (2500 - <4000g) and term gestation (37 weeks or greater). When numbers permitted, associations between specific anomalies and childhood cancer were examined.

Mantel-Haenszel stratified analyses were initially used to describe group characteristics and evaluate confounding. Logistic regression was used to calculate odds ratios (ORs) and 95% confidence interval (CIs) for the evaluation of childhood cancer risk in relation to presence of any anomaly, as well as the presence of specific anomalies. We also estimated the risk of specific cancer types in relation to the presence of any anomaly, and (to the extent possible) in relation to specific type of anomaly. ORs were adjusted for the matching variables of birth year and gender, and for maternal age at delivery (12–19, 20–24, 25–29, 30–34, 35+ years). Other variables considered for their possible effects on the OR included maternal prenatal smoking (yes/no), marital status, race/ethnicity (White, Black, Hispanic, Asian, Native American, Pacific Islander, Other), education (<12, 12, and 13+ years), and type of health insurance. As none of these meaningfully (>10%) altered the ORs, results are adjusted for birth year, sex, and maternal age only. We assessed possible trends of increased risk with increasing numbers of anomalies (0,1,2,3+ and, among those with anomalies only, 1,2,3+; separately for all anomalies and non-chromosomal anomalies) using likelihood ratio tests for adding grouped-linear versions of categorical variables to models including the confounders.

## Results

Childhood cancer cases were more likely than controls to have mothers aged 35 years or older, to be white, or to have a birthweight >4000g (Table 1). The most common types of cancer were leukemia (28%), central nervous system (CNS) tumors (22%), and lymphoma (11%).

**Table 1:** Characteristics of childhood cancer cases and controls born in Washington state, 1984–2013.

	Case (N = 4105) ^a^		Control (N = 45653) ^a^	
Characteristic	n	%	n	%
**Birth year**				
1984–1986	467	11.4	5,153	11.3
1987–1989	548	13.4	5,999	13.1
1990–1994	1,011	24.6	11,581	25.4
1995–1999	809	19.7	9,128	20.0
2000–2004	608	14.8	6,606	14.5
2005–2009	502	12.2	5,433	11.9
2010–2013	160	3.9	1,753	3.8
**Gender**				
Male	2,111	51.6	23,306	51.1
Female	1,984	48.5	22,346	49.0
**Maternal age (years)**				
<20	314	7.7	4,643	10.2
20–24	973	23.8	11,446	25.1
25–29	1,223	29.9	13,589	29.8
30–34	977	23.9	10,504	23.1
35+	600	14.7	5,374	11.8
**Maternal race/ethnicity**				
White	3,243	81.0	34,756	77.9
Black	130	3.3	1,759	3.9
Hispanic	300	7.5	3,934	8.8
Asian	211	5.3	2,376	5.3
Native American	68	1.7	1,016	2.3
Pacific Islander	50	1.3	734	1.7
Other	1	0.0	24	0.1
**Maternal prenatal smoking**				
No	2,995	83.7	32,774	81.8
Yes	585	16.3	7,296	18.2
**Gestational age (weeks)**				
<37	342	8.6	3,449	7.8
37-<42	3,466	86.7	38,790	87.4
42+	191	4.8	2,143	4.8
**Birthweight (g)**				
<2500	233	5.7	2,528	5.6
2500–3999	3,205	78.5	36,980	81.4
4000+	646	15.8	5,952	13.1
**Age at diagnosis (years)**				
<5	1,883	45.9		
5–9	796	19.4	—	—
10-<20	1,426	34.7	—	—
			—	—
**Type of cancer**				
Leukemia	1,140	27.8	—	—
Lymphoma	460	11.2	—	—
CNS	890	21.7	—	—
Neuroblastoma	327	8.0	—	—
Retinoblastoma	110	2.7	—	—
Renal	215	5.2	—	—
Hepatic	63	1.5	—	—
Bone	162	4.0	—	—
Soft-tissue sarcoma	268	6.5	—	—
Germ cell	163	4.0	—	—
Other malignancy	292	7.1	—	—
Unspecified malignancy	15	0.4	—	—

^**a**^ Numbers may not add up to total due to missing data.

A greater proportion of cases (7%) than controls (5%) had at least one major congenital anomaly identified (OR: 1.46, 95% CI 1.28–1.65) (Table 2). The presence of any minor anomaly in the absence of a major anomaly (OR: 0.52, 95% CI 0.24–1.10) or an unspecified anomaly that could not be classified as major or minor (OR: 1.01, 95% CI 0.90–1.14) did not differ markedly in cases and controls. The ORs for childhood cancer increased with increasing numbers of major anomalies, from 1.35 (95% CI 1.17–1.55) for a single anomaly to 2.79 (95% CI 1.44–5.43) for 3 or more anomalies. When only non-chromosomal anomalies were considered in relation to any cancer, the OR remained increased (OR: 1.35, 95% CI 1.18–1.54). A similar pattern was observed after restriction of analyses to children with normal birthweight (2500 -<4000g) with term (37+ weeks) deliveries.

**Table 2:** Presence of congenital anomalies among childhood cancer cases and controls born in Washington state, 1984–2013.

Anomalies present	Case N = 4105		Control N = 45653		OR^a^	95% CI
***Major/Minor Anomalies***	**n**	**%**	**n**	**%**		
Any Anomaly						
No	3,452	84.1	39,400	86.3	1.00	(ref)
Major Anomaly Only	274	6.8	2,208	4.8	1.41	1.24–1.61
Major + Minor Anomalies	20	0.5	84	0.2	2.65	1.62–4.33
Minor Anomalies Only	7	0.2	152	0.3	0.52	0.24–1.10
Unspecified anomaly	352	8.6	3,809	8.3	1.01	0.90–1.14
***Major Anomalies Only*** ^**b**^						
Any Anomaly						
No	3,459	92.2	39,552	94.5	1.00	(ref)
Yes	294	7.8	2,292	5.5	1.46	1.28–1.66
Any Anomaly (Non-Chromosomal) ^c^						
No	3,482	92.9	39,607	94.7	1.00	(ref)
Yes	266	7.1	2,237	5.3	1.35	1.18–1.54
Number of Anomalies ^d^						
0	3,821	93.1	43,431	95.1	1.00	(ref)
1	235	5.7	1,989	4.4	1.35	1.17–1.55
2	38	0.9	190	0.4	2.30	1.62–3.26
3+	11	0.3	43	0.1	2.79	1.44–5.43
Number Non-chromosomal Anomalies ^c^^,^^d^						
0	3,848	93.7	43,469	95.2	1.00	(ref)
1	225	5.5	1,971	4.3	1.29	1.12–1.49
2	22	0.5	178	0.4	1.42	0.91–2.22
3+	10	0.2	35	0.1	3.11	1.54–6.30
**CNS Anomalies**						
Any CNS						
No	4,089	99.6	45,592	99.9	1.00	(ref)
Yes	16	0.4	61	0.1	2.99	1.71–5.19
Spina Bifida						
No	3,729	100.0	41,733	100.0	1.00	(ref)
Yes	1	0.0	14	0.0	0.84	0.11–6.38
Hydrocephalus						
No	2,864	99.7	32,306	99.9	1.00	(ref)
Yes	10	0.3	17	0.1	3.95	1.45–10.74
Microcephalus						
No	2,864	99.9	32,314	100.0	1.00	(ref)
Yes	4	0.1	7	0.0	6.64	1.94–22.75
Other CNS						
No	2,180	100.0	24,621	99.9	1.00	(ref)
Yes	1	0.1	22	0.1	0.37	0.04–3.54
**Heart/Circulatory Anomalies**						
Any Heart						
No	4074	99.2	45,365	99.4	1.00	(ref)
Yes	31	0.8	288	0.6	1.18	0.82–1.72
Any Heart/Circulatory						
No	3,687	98.7	41,426	99.2	1.00	(ref)
Any	7	0.2	45	0.1	1.70	0.76–3.77
Yes; no patent ductus arteriosus (PDA)	30	0.8	204	0.5	1.62	1.10–2.39
Yes; PDA	10	0.3	102	0.2	1.09	0.57–2.08
Other Circulatory						
No	2,177	99.6	24,563	99.6	1.00	(ref)
Yes	9	0.4	103	0.4	0.89	0.41–1.93
**Oral Clefts**						
Any Cleft						
No	4,102	99.9	45,576	99.8	1.00	(ref)
Yes	3	0.1	77	0.2	0.44	0.14–1.38
Cleft Lip/Palate						
No cleft lip/palate	3,726	99.9	41,672	99.8	1.00	(ref)
Cleft lip or palate	0	0.0	53	0.1	—	—
Lip	0	0.0	12	0.0	—	—
Palate	4	0.1	13	0.0	3.36	1.09–10.34
Cleft Palate only						
No	861	99.8	9,422	99.8	1.00	(ref)
Yes	2	0.2	20	0.2	2.84	0.59–13.72
**GI Anomalies**						
Any GI Anomaly						
No	4,086	99.5	45,583	99.8	1.00	(ref)
Yes	19	0.5	70	0.2	3.07	1.85–5.11
Anal Atresia						
No	2,863	99.9	32,311	100.0	1.00	(ref)
Yes	4	0.1	12	0.0	4.75	1.49–15.19
Omphalocele						
No	3,725	99.9	41,729	100.0	1.00	(ref)
Yes	5	0.1	19	0.0	3.12	1.16–8.38
Gastroschisis						
No	862	99.8	9,422	99.9	1.00	(ref)
Yes	2	0.2	9	0.1	3.18	0.67–15.16
Other Gastrointestinal Anomalies						
No	2,171	99.5	24,608	99.8	1.00	(ref)
Yes	12	0.5	39	0.2	3.41	1.73–6.71
**Chromosomal Anomalies** ^c^						
Any Chromosomal Anomaly						
No	4,055	98.8	45,582	99.8	1.00	(ref)
Yes	50	1.2	71	0.2	7.52	5.21–10.84
Downs Syndrome						
No	3,695	98.9	41,708	99.9	1.00	(ref)
Yes	42	1.1	40	0.1	10.86	7.02–16.81
Other Chromosomal Anomaly						
No	3,035	99.7	34,031	99.9	1.00	(ref)
Yes	9	0.3	33	0.1	3.03	1.44–6.35
**Genitourinary (GU) Anomalies**						
Any GU Anomaly						
No	4,067	99.1	45,300	99.2	1.00	(ref)
Yes	38	0.9	353	0.8	1.19	0.85–1.66
Hypospadias						
No	444	100.0	4,869	99.8	1.00	(ref)
Yes	0	0.0	12	0.2	—	—
Renal Agenesis						
No	2,180	100.0	24,628	99.9	1.00	(ref)
Yes	1	0.0	14	0.1	1.08	0.14–8.45
Other Urogenital Anomalies						
No	2,161	98.4	24,429	98.8	1.00	(ref)
Yes	35	1.6	292	1.2	1.26	0.86–1.85
**Musculoskeletal Anomalies**						
Any Musculoskeletal Anomaly						
No	4,055	98.8	45,232	99.1	1.00	(ref)
Yes	50	1.2	421	0.9	1.33	0.99–1.79
Any Musculoskeletal Anomaly (hip displacement excluded)						
No	4,066	99.0	45,289	99.2	1.00	(ref)
Yes	39	1.0	364	0.8	1.20	0.86–1.68
Musculoskeletal Anomaly						
No	2,827	98.1	32007	98.7	1.00	(ref)
Any	39	1.4	349	1.1	1.30	0.91–1.86
Congenital Hip Displacement	14	0.5	62	0.2	2.49	1.39–4.48
Limb Reduction	0	0.0	6	0.0	—	—
Other Limb Reduction	0	0.0	4	0.0	—	—
Any dactyly						
No	4,094	99.7	45,557	99.8	1.00	(ref)
Yes	11	0.3	96	0.2	1.29	0.69–2.42
Dactyly						
No	2,860	99.6	32,254	99.7	1.00	(ref)
Yes (any)	2	0.1	23	0.1	0.99	0.23–4.20
Adactyly	0	0.0	6	0.0	—	—
Polydactyly	5	0.2	41	0.1	1.91	0.71–5.12
Syndactyly	4	0.1	26	0.1	2.08	0.70–6.18
Club Foot						
No	2,865	99.9	32,291	99.9	1.00	(ref)
Yes	3	0.1	31	0.1	0.77	0.18–3.22
Limb Reduction (any)						
No	863	100.0	9,425	99.8	1.00	(ref)
Yes	0	0.0	15	0.2	—	—
**Anomalies of the Integument**						
Any Skin Anomaly						
No	4,044	98.5	44,987	98.5	1.00	(ref)
Yes	61	1.5	666	1.5	1.03	0.79–1.34
**Other Anomalies**						
Other Anomalies						
No	2,821	98.1	31,884	98.5	1.00	(ref)
Yes	56	1.9	477	1.5	1.26	0.94–1.69

^a^ Adjusted for birth year, sex, and maternal age.

^b^ Referent group includes children with minor anomalies. Excludes 352 cases and 3809 controls with unspecified anomalies that could not be classified as major or minor. Except for where indicated, chromosomal anomalies were not included within each malformation type.

^c^ Chromosomal anomalies include Downs (68% of all chromosomal anomalies); Patau’s (3%), Edward’s (1.6%), Klinefelter’s (1.6%), and Turner’s syndrome/gonadal dysgenesis (2.5%); autosomal anomalies not elsewhere classified, and various other conditions due to chromosome anomalies. Children may have multiple chromosomal anomalies, therefore disaggregated numbers may exceed the total number of children with these birth defects.

^d^ Trend test p<0.0001 for any, and non-chromosomal anomalies among all subjects. Among subjects with anomalies only, p = 0.003 for number of any anomalies; p = 0.077 for number of non-chromosomal anomalies.

Increased ORs for childhood cancer were observed for all anomalies, with the exception of oral clefts (OR: 0.56, 95% CI 0.20–1.52), club foot (OR: 0.77, 95% CI: 0.18–3.22), dactyly (OR: 0.99, 95% CI: 0.23–4.20), spina bifida (OR: 0.84, 95% CI: 0.11–6.38), other CNS anomalies (OR: 0.37; 95% CI: 0.04–3.54), and other circulatory anomalies (OR: 0.89, 95% CI: 0.41–1.93). The effect sizes varied: the greatest OR was observed for chromosomal anomalies (OR: 7.52, 95% CI 5.21–10.84). Large and positive ORs were also associated with gastrointestinal anomalies (OR: 3.07, 95% CI 1.85–5.11) and CNS anomalies (OR 2.99, 95% CI: 1.71–5.19). Within general anomalies, selected specific conditions had increased ORs, including microcephalus (OR: 6.64, 95% CI 1.94–22.75), hydrocephalus (OR: 3.95, 95% CI 1.45–10.74), anal atresia (OR: 4.75, 95% CI 1.49–15.19), and Down syndrome (OR: 10.86, 95% CI 7.02–16.81).

ORs were increased for the association of anomalies in relation to childhood cancer diagnosed in all age groups, although the magnitudes of the associations were greatest for cancers diagnosed at <5 years of age (Table 3). Modestly increased ORs with CIs including one were noted for cancer diagnosed between 5–9 years of age, although statistically significant associations were noted for cancers diagnosed in the older (10–19 years) age group.

**Table 3:** Odds ratios for childhood cancer diagnosed at different ages in relation to the presence of major anomalies among children born in Washington State, 1984–2013.

	Controls ^a^	Cases ^a^ *Age at diagnosis (years)*					
	N = 41,844	<5 (N = 1,740)		5–9 (N = 729)		10–19 (N = 1,279)	
Exposure	n (%)	n (%)	OR^b^ (95% CI)	n (%)	OR^b^ (95% CI)	n (%)	OR^b^ (95% CI)
Major anomaly	2,292 (5.5)	161 (9.2)	1.75 (1.48–2.07)	43 (5.9)	1.09 (0.80–1.49)	90 (7.0)	1.29 (1.03–1.61)
Major non-chromosomal anomaly	2,237 (5.4)	137 (7.9)	1.51 (1.26–1.81)	43 (5.9)	1.12 (0.82–1.53)	86 (6.7)	1.27 (1.02–1.60)

^a^ Excludes children with unknown major/minor anomaly status

^b^ Adjusted for birth year, maternal age, and sex

**Fig 1:**
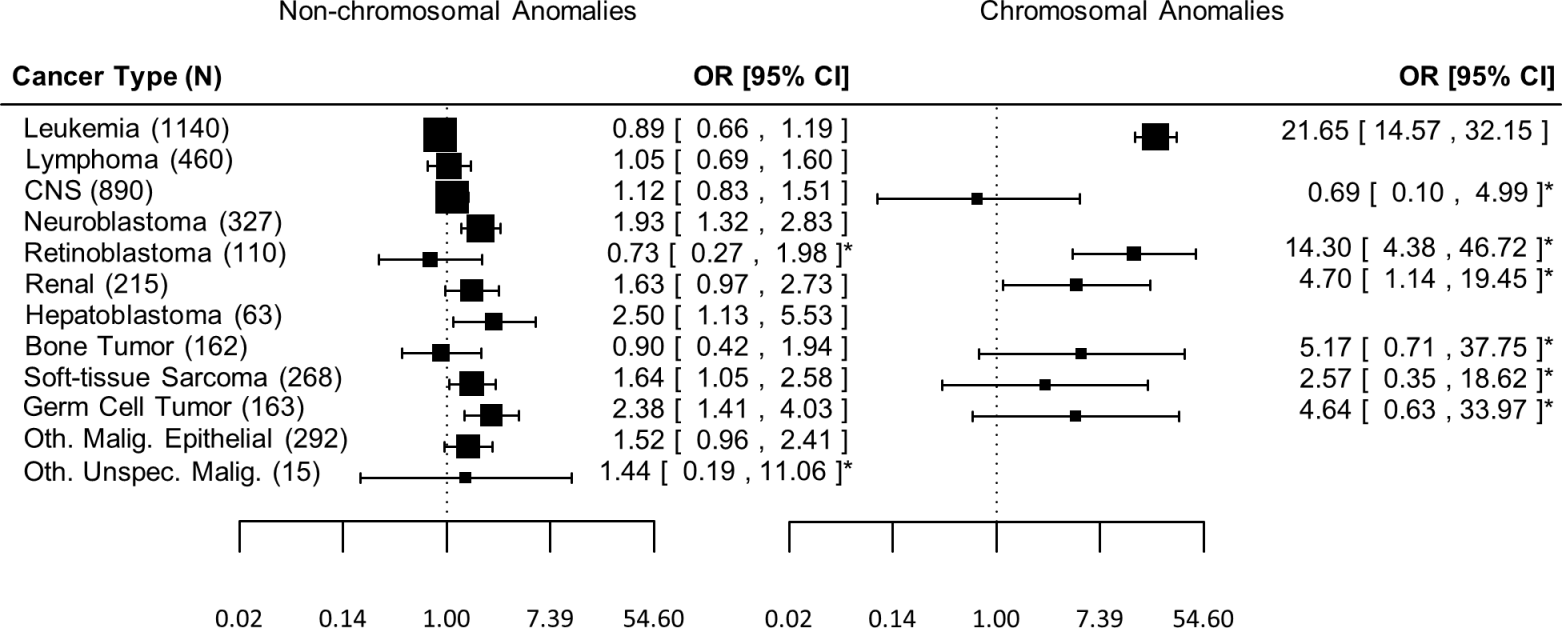
Odds Ratios (OR) for the associations of specific childhood cancer types in relation to presence of major non-chromosomal and chromosomal anomalies, among children born in Washington State, 1984–2013. Estimates adjusted for birth year, sex, and maternal age. Non-chromosomal anomalies results exclude individuals with concurrent chromosomal anomalies. *Indicates number of cases <5.

The presence of a chromosomal anomaly was generally associated with greater ORs for most types of cancer than was the presence of non-chromosomal anomalies (Fig 1). Non-chromosomal anomalies were associated with greater than two-fold increased risk of hepatoblastoma (OR: 2.50, 95% CI 1.13–5.53) and germ cell tumors (OR: 2.38, 95% CI 1.41–4.03), but also with increased risk for neuroblastoma (OR: 1.93, 95% CI 1.32–2.83) and soft-tissue sarcomas (OR: 1.71, 95% CI 1.10–2.65). The presence of a chromosomal anomaly was associated with large increased risk for leukemia (OR: 21.65, 95% CI: 14.57–32.15), retinoblastoma (OR: 14.30, 95% CI: 4.38–46.72), and renal tumors (OR: 4.70, 95% CI 1.14–19.45). Increased ORs for all other cancer types examined except CNS tumors in relation to chromosomal anomalies were also observed, although the estimates were imprecise and confidence intervals included one.

**Fig 2:**
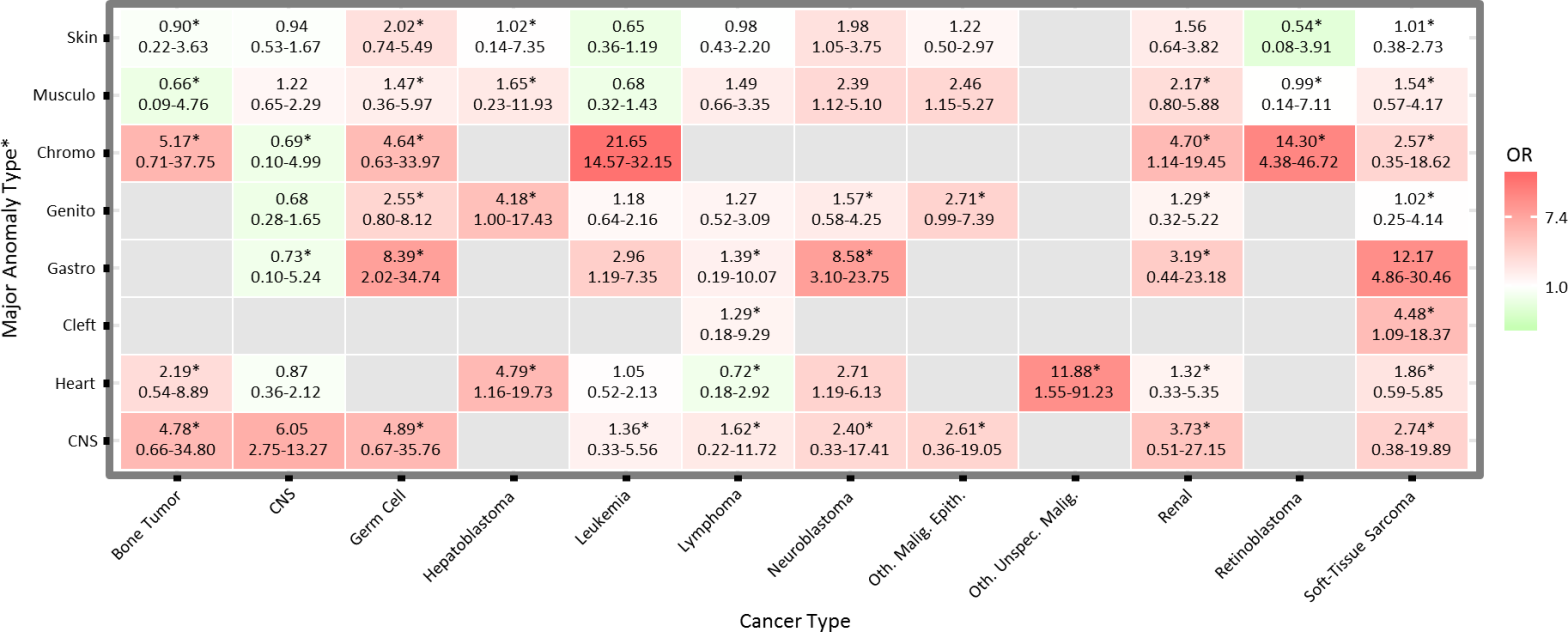
Odds ratios for the associations of major anomaly types in relation to types of childhood cancer among children born in Washington State, 1984–2013. Estimates adjusted for birth year, sex, and maternal age. Non-chromosomal anomaly results exclude individuals with concurrent chromosomal anomalies. *Indicates number of cases <5.

We explored the associations of specific anomaly types in relation to specific types of childhood cancer (Fig 2). The largest ORs were observed for the presence of chromosomal anomalies in relation to leukemia (OR: 21.65, 95% CI 14.57–32.15) and retinoblastoma (OR: 14.30, 95% CI 4.38–46.72), and for the presence of gastrointestinal anomalies in relation to soft-tissue sarcoma (OR: 12.17, 95% CI 4.86–30.46). CNS tumors were associated with CNS anomalies (OR: 6.05, 95% CI 2.75–13.27) but not with other anomalies. Most other non-chromosomal anomalies were associated with several types of cancer. The presence of a gastrointestinal anomaly was associated with increased ORs for germ cell, leukemia, neuroblastoma, and soft-tissue sarcoma. Heart anomalies were associated with hepatoblastoma, neuroblastoma, and other unspecified malignancies.

## Discussion

Congenital anomalies have been associated with childhood cancer in several prior studies. Our observed overall increased risk for cancer in relation to congenital anomalies is consistent with results of other U.S. population-based data linkage studies based on data from California,[6] Texas,[2] Oklahoma,[5] and a pooled analysis of data from three other states (Utah, Arizona, and Iowa, i.e., UTAZIA).[7] Similar results have been reported in population-based health registry studies in Australia,[15] Canada,[16] the United Kingdom,[17] and Norway and Sweden.[18] While the association of chromosomal anomalies with childhood cancer occurrence has been fairly well established in previous population-based data linkage studies, with population-based studies reporting estimates of >10-fold increased risk.[2, 6, 7], the majority of anomalies are non-chromosomal in origin. Our study lends to the growing body of evidence that non-chromosomal anomalies are also associated with childhood cancer risk.[2, 5–7] Additionally, our results indicate that an increasing number of non-chromosomal anomalies was more strongly associated with increased cancer risk compared to those children with only one non-chromosomal anomaly (OR for three or more anomalies: 3.11, 95% CI: 1.54–6.11 vs. OR for one anomaly: 1.29, 95% CI: 1.12–1.49). This may suggest that children with previously unidentified multiple malformation syndromes (and no obvious chromosomal anomaly) may be at a significant risk of cancer.

Our data confirm the association between chromosomal anomalies and childhood cancer, including the well documented association of Down syndrome and acute leukemia. Although there have been efforts to identify factors associated with acute lymphoblastic leukemia in children with Down syndrome (e.g., maternal health conditions and irradiation), most results have been null.[19, 20] Our results also support an association between having a chromosomal anomaly with risk of retinoblastoma, which is consistent with other studies.[2] It is likely the primary driver behind this association is an autosomal deletion of 13q14, which includes the *RB1* gene, a germline predisposition gene for retinoblastoma.[21] We also observed an association of chromosomal anomalies with renal tumors (e.g., Wilms tumor). Notably, Wilms tumor, aniridia, genitourinary anomalies, and mental retardation (i.e., WAGR syndrome) are a set of conditions associated with a deletion on 11p13, which includes the *WT1* gene.[22]

Our results indicate an increased risk of childhood cancer in relation to presence of anomalies for cancers diagnosed in all age groups. When only non-chromosomal anomalies were considered, the risk estimates generally remained increased, supporting results of other studies indicating an association with childhood cancer diagnosed at different ages, but with a slight decrease in risk with attained age.[5] We also observed increased cancer risk in relation to increasing number of anomalies present, which is consistent with one earlier report.[18] Also consistent with other population-based data linkage studies that examined non-chromosomal anomalies, we observed increased risks of selected cancer types. We found associations between having major non-chromosomal anomalies and increased risks of neuroblastoma, hepatoblastoma, soft-tissue sarcoma, and germ cell tumors. With the exception of leukemia, retinoblastoma, and bone tumors, we observed increased risks among all other cancer types however these associations were not statistically significant.

Notably, our study supports other recent population-based registry linkage studies in demonstrating the relationship between non-chromosomal anomalies and childhood cancer.[5–7] Our overall effect estimate for the risk of cancer among children with non-chromosomal anomalies (OR = 1.35) was only slightly attenuated compared to those reported by Janitz et al. (HR = 2.50)[5], Botto et al. (incidence rate ratio [IRR] = 2.00),[7] and Fisher et al. (HR = 1.58).

We did note specific cancer types associated with having non-chromosomal anomalies. For example, the OR for neuroblastoma in relation to non-chromosomal anomalies (OR = 1.90) was largely consistent with two previous U.S. registry linkage studies evaluating the risk of this malignancy in children with non-chromosomal anomalies (HR = 2.85[6] and IRR = 2.21[7]). Also, we observed a positive association of non-chromosomal anomalies with hepatoblastoma, consistent with the only other U.S. registry linkage study (UTAZIA study) to evaluate this particular relationship, although the effect size was larger in that assessment (IRR = 14.47 vs. our OR = 2.45).[7] Having a non-chromosomal anomaly was associated with soft-tissue sarcomas, consistent with the one U.S. registry linkage study evaluating this specific relationship.[5] Our observed association with renal tumors (OR = 1.71) was stronger than reported in the California (HR = 1.45) and UTAZIA (IRR = 1.03) studies.[6, 23] Finally, our observation of a positive association between germ cell tumors and non-chromosomal anomalies supports results of other studies that were able to assess this association.[5, 6, 23]

In our assessment, non-chromosomal anomalies overall were not strongly associated with leukemia, lymphoma or CNS tumors, consistent with results of the other U.S. registry linkage studies.[5, 6, 23], despite differences in birth defect surveillance across studies. (Washington does not have an active birth defects surveillance program as in California, Utah, Iowa, and Oklahoma.) Notably, the prevalence of congenital anomalies was slightly higher in our assessment (5.5%) when compared to these states (e.g., ~4% [2, 5]), however, the ascertainment of anomalies was independent of case status in our assessment, and therefore uniform for those children who did and did not develop cancer, which reduces the likelihood of differential misclassification.

Aside from an association of CNS anomalies with CNS tumors (an association that may be due to reverse causation given the majority of these anomalies were hydrocephalus-related), there was no strong evidence that non-chromosomal anomalies were likely to be specifically associated with childhood cancer occurring in the same organ system or anatomic location, although our ability to investigate this was limited by small numbers. Although neuroblastoma was associated with heart and gastrointestinal anomalies, it was also associated with musculoskeletal and skin anomalies. Few studies have been able to examine associations of specific non-chromosomal anomalies with specific cancer types, but of these, a generally consistent finding is an association of CNS defects with CNS tumors,[8, 18, 24] as we observed.

An important strength of our study was use of linked population-based health registry data, allowing us to avoid some biases that may be present in clinic-based or interview studies. We also increased the sensitivity of birth defect ascertainment by utilizing specific diagnostic codes in addition to birth record information. We were able to examine specific congenital anomalies. Our study must also be considered in the light of certain limitations. In order to identify major and minor anomalies, we used the classification system developed by Rasmussen and colleagues,[13] which utilizes CDC-BPA codes that are more specific than the ICD-9 codes available to us for this study. Despite our ability to utilize linked hospital discharge records, our ascertainment of anomalies is likely less complete than for studies using data from active birth defects surveillance programs.[5, 15–17] However, several birth defects surveillance programs only monitor specific anomalies, whereas we evaluated all congenital anomalies. It is also possible that some children in the control group may have moved out-of-state and been diagnosed with cancer elsewhere, however as childhood cancer is quite rare, any effect of this is likely minimal and would bias towards the null. Because of the availability of birth and cancer registry data during different time periods, some children diagnosed in earlier study years at older ages would not have been included, however sensitivity analyses restricting subjects to only those with similar opportunity (e.g., at least 5 years; at least 10 years) to have been identified in the cancer registry did not substantially alter results (S2 Table). We were also limited in our ability to evaluate possible associations with minor anomalies which may not be detected until later in a child’s life, and would not appear on birth certificates or hospital discharge records for the birth hospitalization. Finally, children with some types of congenital anomalies may die prematurely and therefore lack the opportunity to develop childhood cancer, which could possibly attenuate our associations.[5]

The etiologies of most non-chromosomal anomalies are largely unknown,[1] despite evidence that factors such as maternal obesity, prenatal smoking, and some chemical or environmental exposures may increase the occurrence of certain defect types.[1, 25] Our knowledge of childhood cancer causes is similarly limited, with few recognized external etiologies (e.g., ionizing radiation) although common variation and intrinsic factors such as birthweight and parental age are consistently associated with childhood cancers. Identification of factors associated with progression from defect presence to cancer occurrence, or of shared pathways (genetic and environmental) for both conditions may elucidate potential mechanisms to modify cancer risk. Future assessments should include pooling efforts across multiple regions. This will optimize our ability to identify associations between specific congenital anomalies and specific cancers. The ultimate goal of this work would be to inform screening strategies for children at high risk of developing cancer.

## Supporting information

S1 Table Major and minor congenital malformation ascertainment based on birth certificates and hospital discharge record for the infant’s birth.[13] (DOCX)

S2 Table Major anomalies among childhood cancer cases diagnosed by ages 5 or 10 years, and their controls, by selected time periods. (DOCX)

## References

[1] Christianson A, Howson C, Modell B. Global Report on Birth Defects: The Hidden Toll of Dying and Disabled Children. 2006.

[2] Carozza SE, Langlois PH, Miller EA, Canfield M. Are children with birth defects at higher risk of childhood cancers? Am J Epidemiol. 2012;175(12):1217–24. Epub 2012/04/27. doi: 10.1093/aje/kwr470 . 22534203 10.1093/aje/kwr470

[3] Hasle H, Clemmensen IH, Mikkelsen M. Risks of leukaemia and solid tumours in individuals with Down's syndrome. Lancet. 2000;355(9199):165–9. doi: 10.1016/S0140-6736(99)05264-2 10675114 10.1016/S0140-6736(99)05264-2

[4] Motegi T, Kaga M, Yanagawa Y, Kadowaki H, Watanabe K, Inoue A, et al A recognizable pattern of the midface of retinoblastoma patients with interstitial deletion of 13q. Hum Genet. 1983;64(2):160–2. . 6885051 10.1007/BF00327116

[5] Janitz AE, Neas BR, Campbell JE, Pate AE, Stoner JA, Magzamen SL, et al Childhood cancer in children with congenital anomalies in Oklahoma, 1997 to 2009. Birth Defects Res A Clin Mol Teratol. 2016 Epub 2016/03/08. doi: 10.1002/bdra.23494 . 26945683 10.1002/bdra.23494 PMC4946965

[6] Fisher PG, Reynolds P, Von Behren J, Carmichael SL, Rasmussen SA, Shaw GM. Cancer in children with nonchromosomal birth defects. J Pediatr. 2012;160(6):978–83. Epub 2012/01/17. doi: 10.1016/j.jpeds.2011.12.006 ; PubMed Central PMCID: PMCPMC4490790. 22244463 10.1016/j.jpeds.2011.12.006 PMC4490790

[7] Botto LD, Flood T, Little J, Fluchel MN, Krikov S, Feldkamp ML, et al Cancer risk in children and adolescents with birth defects: a population-based cohort study. PLoS One. 2013;8(7):e69077 Epub 2013/07/23. doi: 10.1371/journal.pone.0069077 ; PubMed Central PMCID: PMCPMC3714243. 23874873 10.1371/journal.pone.0069077 PMC3714243

[8] Narod SA, Hawkins MM, Robertson CM, Stiller CA. Congenital anomalies and childhood cancer in Great Britain. Am J Hum Genet. 1997;60(3):474–85. Epub 1997/03/01. ; PubMed Central PMCID: PMCPMC1712528. 9042906 PMC1712528

[9] Lydon-Rochelle MT, Holt VL, Cardenas V, Nelson JC, Easterling TR, Gardella C, et al The reporting of pre-existing maternal medical conditions and complications of pregnancy on birth certificates and in hospital discharge data. Am J Obstet Gynecol. 2005;193(1):125–34. Epub 2005/07/16. doi: 10.1016/j.ajog.2005.02.096 . 16021070 10.1016/j.ajog.2005.02.096

[10] Lydon-Rochelle MT, Holt VL, Nelson JC, Cardenas V, Gardella C, Easterling TR, et al Accuracy of reporting maternal in-hospital diagnoses and intrapartum procedures in Washington State linked birth records. Paediatr Perinat Epidemiol. 2005;19(6):460–71. Epub 2005/11/05. doi: 10.1111/j.1365-3016.2005.00682.x . 16269074 10.1111/j.1365-3016.2005.00682.x

[11] Parrish KM, Holt VL, Connell FA, Williams B, LoGerfo JP. Variations in the accuracy of obstetric procedures and diagnoses on birth records in Washington State, 1989. Am J Epidemiol. 1993;138(2):119–27. Epub 1993/07/15. . 8342530 10.1093/oxfordjournals.aje.a116834

[12] Northam S, Knapp TR. The reliability and validity of birth certificates. J Obstet Gynecol Neonatal Nurs. 2006;35(1):3–12. Epub 2006/02/10. doi: 10.1111/j.1552-6909.2006.00016.x . 16466348 10.1111/j.1552-6909.2006.00016.x

[13] Rasmussen SA, Olney RS, Holmes LB, Lin AE, Keppler-Noreuil KM, Moore CA. Guidelines for case classification for the National Birth Defects Prevention Study. Birth Defects Res A Clin Mol Teratol. 2003;67(3):193–201. Epub 2003/06/12. doi: 10.1002/bdra.10012 . 12797461 10.1002/bdra.10012

[14] Steliarova-Foucher E, Stiller C, Lacour B, Kaatsch P. International Classification of Childhood Cancer, third edition. Cancer. 2005;103(7):1457–67. Epub 2005/02/16. doi: 10.1002/cncr.20910 . 15712273 10.1002/cncr.20910

[15] Altmann AE, Halliday JL, Giles GG. Associations between congenital malformations and childhood cancer. A register-based case-control study. Br J Cancer. 1998;78(9):1244–9. Epub 1998/11/20. ; PubMed Central PMCID: PMCPMC2062998. 9820188 10.1038/bjc.1998.662 PMC2062998

[16] Agha MM, Williams JI, Marrett L, To T, Zipursky A, Dodds L. Congenital abnormalities and childhood cancer. Cancer. 2005;103(9):1939–48. Epub 2005/03/17. doi: 10.1002/cncr.20985 . 15770693 10.1002/cncr.20985

[17] Rankin J, Silf KA, Pearce MS, Parker L, Ward Platt M. Congenital anomaly and childhood cancer: A population-based, record linkage study. Pediatr Blood Cancer. 2008;51(5):608–12. Epub 2008/07/16. doi: 10.1002/pbc.21682 . 18623214 10.1002/pbc.21682

[18] Bjorge T, Cnattingius S, Lie RT, Tretli S, Engeland A. Cancer risk in children with birth defects and in their families: a population based cohort study of 5.2 million children from Norway and Sweden. Cancer Epidemiol Biomarkers Prev. 2008;17(3):500–6. Epub 2008/02/26. doi: 10.1158/1055-9965.EPI-07-2630 . 18296646 10.1158/1055-9965.EPI-07-2630

[19] Linabery AM, Olshan AF, Gamis AS, Smith FO, Heerema NA, Blair CK, et al Exposure to medical test irradiation and acute leukemia among children with Down syndrome: a report from the Children's Oncology Group. Pediatrics. 2006;118(5):e1499–e508. doi: 10.1542/peds.2006-0644 17030598 10.1542/peds.2006-0644

[20] Ognjanovic S, Puumala S, Spector LG, Smith FO, Robison LL, Olshan AF, et al Maternal health conditions during pregnancy and acute leukemia in children with Down syndrome: A Children's Oncology Group study. Pediatr Blood Cancer. 2009;52(5):602–8. Epub 2009/01/17. doi: 10.1002/pbc.21914 ; PubMed Central PMCID: PMC2659730. 19148952 10.1002/pbc.21914 PMC2659730

[21] Bunin GR, Emanuel BS, Meadows AT, Buckley JD, Woods WG, Hammond GD. Frequency of 13q abnormalities among 203 patients with retinoblastoma. J Natl Cancer Inst. 1989;81(5):370–4. . 2915374 10.1093/jnci/81.5.370

[22] Riccardi VM, Sujansky E, Smith AC, Francke U. Chromosomal imbalance in the Aniridia-Wilms' tumor association: 11p interstitial deletion. Pediatrics. 1978;61(4):604–10. . 208044

[23] Botto LD, Khoury MJ, Mastroiacovo P, Castilla EE, Moore CA, Skjaerven R, et al The spectrum of congenital anomalies of the VATER association: an international study. American journal of medical genetics. 1997;71(1):8–15. Epub 1997/07/11. . 9215761 10.1002/(sici)1096-8628(19970711)71:1<8::aid-ajmg2>3.0.co;2-v

[24] Fischer M, Schwieger M, Horn S, Niebuhr B, Ford A, Roscher S, et al Defining the oncogenic function of the TEL/AML1 (ETV6/RUNX1) fusion protein in a mouse model. Oncogene. 2005;24(51):7579–91. doi: 10.1038/sj.onc.1208931 16044150 10.1038/sj.onc.1208931

[25] Madsen NL, Schwartz SM, Lewin MB, Mueller BA. Prepregnancy body mass index and congenital heart defects among offspring: a population-based study. Congenit Heart Dis. 2013;8(2):131–41. Epub 2012/09/13. doi: 10.1111/j.1747-0803.2012.00714.x . 22967199 10.1111/j.1747-0803.2012.00714.x

