# Supplementary material for: Childhood cancer risk in those with chromosomal and non-chromosomal congenital anomalies in Washington State: 1984-2013
Source: PLoS One. 2017 Jun 8;12(6):e0179006. doi: 10.1371/journal.pone.0179006 (PMC5464621; doi:10.1371/journal.pone.0179006)
Supplement: S1 Table — (DOCX) [file pone.0179006.s001.docx]

**S1 Table. Major and minor congenital malformation ascertainment based on birth certificates and ICD-9 diagnosis codes in the hospital discharge record for the infant’s birth.**

| **Malformation** | **ICD-9-CM Code** | **Major** | **Minor** | **Birth Certificate Designation**  **(if relevant)** |
| --- | --- | --- | --- | --- |
| ***Any Malformation*** | ***740 – 759*** | ✓ | ✓ | Any Malformation |
| **CNS Malformations** | **740 – 742** |  |  |  |
| ***Any CNS Malformation*** | ***740 – 742*** | ✓ |  |  |
| Anencephalus | 740.0 | ✓ |  | Anencephaly |
| Craniorachischisis | 740.1 | ✓ |  |  |
| Iniencephaly | 740.2 | ✓ |  |  |
| Spina bifida with hydrocephalus | 741.0 | ✓ |  | Spina bifida + Hydrocephaly |
| Spina bifida without hydrocephalus | 741.9 | ✓ |  | Spina bifida |
| Encephalocele | 742.0 | ✓ |  |  |
| Microcephalus | 742.1 | ✓ |  | Microcephaly |
| Reduction deformities of brain | 742.2 | ✓ |  |  |
| Congenital hydrocephalus | 742.3 | ✓ |  | Hydrocephaly |
| Non-Specific: | 742.4, 742.59, 742.8, 742.9 | ✓ |  |  |
| ***Other CNS Malformation*** | ***740 – 742*** | ✓ |  | Other CNS Malformations |
| **Eye Malformations** | **743** |  |  |  |
| Cystic eyeball, congenital | 743.03 | ✓ |  |  |
| Cryptophthalmos | 743.06 | ✓ |  |  |
| Simple microphthalmos | 743.11 |  | ✓ |  |
| Simple bupthalmos | 743.21 | ✓ |  |  |
| Capsular and subcapsular cataract | 743.31 | ✓ |  |  |
| Cortical and zonular cataract | 743.32 | ✓ |  |  |
| Nuclear cataract | 743.33 | ✓ |  |  |
| Total and subtotal cataract | 743.34 | ✓ |  |  |
| Congenital aphakia | 743.35 | ✓ |  |  |
| Anomalies of lens shape | 743.36 | ✓ |  |  |
| Congenital ectopic lens | 743.37 | ✓ |  |  |
| Anomalies of corneal size and shape | 743.41 | ✓ |  |  |
| Corneal opacities, interfering with vision | 743.42 | ✓ |  |  |
| Anirida | 743.45 | ✓ |  |  |
| Vitreous anomalies | 743.51 | ✓ |  |  |
| Fundus coloboma | 743.52 | ✓ |  |  |
| Chororetinal degeneration | 743.53 | ✓ |  |  |
| Congenital folds and cysts of posterior segment | 743.54 | ✓ |  |  |
| Congenital macular changes | 743.55 | ✓ |  |  |
| Vascular anomalies | 743.58 | ✓ |  |  |
| Congenital ptosis | 743.61 | ✓ |  |  |
| Congenital deformities of eyelids | 743.62 | ✓ |  |  |
| **Ear, Face, Neck Malformations** | **744** |  |  |  |
| Absence of external ear | 744.01 | ✓ |  |  |
| Anomaly of middle ear, except ossicles | 744.03 | ✓ |  |  |
| Anomalies of ear ossicles | 744.04 | ✓ |  |  |
| Anomalies of inner ear | 744.05 | ✓ |  |  |
| Accessory auricle | 744.1 | ✓ |  |  |
| Absence of ear lobe, congenital | 744.21 | ✓ |  |  |
| Macrotia | 744.22 |  | ✓ |  |
| Microtia | 744.23 |  | ✓ |  |
| Branchial cleft sinus or fistula | 744.41 | ✓ |  |  |
| **Malformation** | **ICD-9-CM Code** | **Major** | **Minor** | **Birth Certificate Designation**  **(if relevant)** |
| Branchial cleft cyst | 744.42 | ✓ |  |  |
| Cervical auricle | 744.43 | ✓ |  |  |
| Preauricular sinus or fistula | 744.46 | ✓ |  |  |
| Preauricular cyst | 744.47 | ✓ |  |  |
| Webbing of neck | 744.5 |  | ✓ |  |
| Macrocheilia | 744.81 |  | ✓ |  |
| Microcheilia | 744.82 |  | ✓ |  |
| Macrostomia | 744.83 | ✓ |  |  |
| Microstomia | 744.84 | ✓ |  |  |
| Non-Specific: | 743.00, 743.10, 743.12, 743.20, 743.22, 743.30, 743.39, 743.43, 743.44, 743.46, 743.47, 743.48, 743.49, 743.56, 743.57, 743.59, 743.63, 743.64, 743.65, 743.66, 743.69, 743.8, 743.9, 744.00, 744.02, 744.09, 744.24, 744.29, 744.3, 744.49, 744.89 | ✓ |  |  |
| Non-Specific Codes (minor): | 744.9 |  | ✓ |  |
| **Heart Malformations** |  |  |  |  |
| ***Any Heart/RespiratoryMalformation*** | ***745 – 747*** | ✓ | ✓ |  |
| ***Any Heart/RespiratoryMalformation (no PDA)*** | ***745 – 747*** | ✓ | ✓ |  |
| ***Heart Malformations***  ***1 – any heart malf***  ***2 – any heart malf (no PDA)***  ***3 – PDA only*** *(Minor)* | ***745 – 747*** | ✓ | ✓ | Heart Malformations |
| Common truncus | 745.0 | ✓ |  |  |
| Complete tranposition of great vessels | 745.10 | ✓ |  |  |
| Double outlet right ventricle | 745.11 | ✓ |  |  |
| Corrected transposition of great vessels | 745.12 | ✓ |  |  |
| Tetralogy of Fallot | 745.2 | ✓ |  |  |
| Common ventricle | 745.3 | ✓ |  |  |
| Ventricular septal defect | 745.4 | ✓ |  |  |
| Ostium secundum type atrial septal defect | 745.5 | ✓ |  |  |
| Ostium primum defect | 745.61 | ✓ |  |  |
| Non-Specific: | 745.19, 745.60, 745.69, 745.8, 745.9 | ✓ |  |  |
| **Other Congenital Anomalies of Heart** | **746** |  |  |  |
| Atresia, congenital | 746.01 | ✓ |  |  |
| Stenosis, congenital | 746.02 | ✓ |  |  |
| Tricuspid atresia and stenosis, congenital | 746.1 | ✓ |  |  |
| Ebstein’s anomaly | 746.2 | ✓ |  |  |
| Congenital stenosis of aortic valve | 746.3 | ✓ |  |  |
| Congenital insufficiency of aortic valve | 746.4 | ✓ |  |  |
| Congenital mitral stenosis | 746.5 | ✓ |  |  |
| Congenital mitral insufficiency | 746.6 | ✓ |  |  |
| Hypoplastic left heart syndrome | 746.7 | ✓ |  |  |
| Subaortic stenosis | 746.81 | ✓ |  |  |
| Cor triatriatum | 746.82 | ✓ |  |  |
| Infundibular pulmonic stenosis | 746.83 | ✓ |  |  |
| Obstructive heart anomalies, NEC | 746.84 | ✓ |  |  |
| Coronary artery anomaly | 746.85 | ✓ |  |  |
| Congenital heart block | 746.86 | ✓ |  |  |
| Malposition of heart and cardiac apex | 746.87 | ✓ |  |  |
| Non-Specific: | 746.00, 746.09, 746.89, 746.9 | ✓ |  |  |
| **Other Congenital Anomalies of Circulatory System** | **747** |  |  |  |
| ***Other Circulatory/RespiratoryMalformations*** | **747 – 748** | ✓ | ✓ | Other Circulatory/Respiratory Anomalies |
| Patent ductus arteriosus | 747.0 |  | ✓ |  |
| Coarctation of aorta | 747.10 | ✓ |  |  |
|  |  |  |  |  |
| **Malformation** | **ICD-9-CM Code** | **Major** | **Minor** | **Birth Certificate Designation**  **(if relevant)** |
| Interruption of aortic arch | 747.11 | ✓ |  |  |
| Anomalies of aortic arch | 747.21 | ✓ |  |  |
| Atresia and stenosis of aorta | 747.22 | ✓ |  |  |
| Anomalies of pulmonary artery | 747.3 | ✓ |  |  |
| Total anomalous pulmonary venous connection | 747.41 | ✓ |  |  |
| Partial anomalous pulmonary venous connection | 747.42 | ✓ |  |  |
| Absence or hypoplasia of umbilical artery | 747.5 |  | ✓ |  |
| Gastrointestinal vessel anomaly | 747.61 |  | ✓ |  |
| Renal vessel anomaly | 747.62 |  | ✓ |  |
| Upper limb vessel anomaly | 747.63 |  | ✓ |  |
| Lower limb vessel anomaly | 747.64 |  | ✓ |  |
| Anomalies of cerebrovascular system | 747.81 |  | ✓ |  |
| Spinal vessel anomaly | 747.82 |  | ✓ |  |
| Non-Specific: | 747.20, 747.29, 747.40, 747.49, 747.60, 747.69, 747.89, 747.9 | ✓ |  |  |
| **Congenital Anomalies of Respiratory System** | **748** |  |  |  |
| Choanal atresia | 748.0 | ✓ |  |  |
| Web of larynx | 748.2 | ✓ |  |  |
| Congenital cystic lung | 748.4 | ✓ |  |  |
| Agenesis, hypoplasia, and dysplasia of lung | 748.5 | ✓ |  |  |
| Non-Specific: | 748.1, 748.3, 748.60, 748.69, 748.8, 748.9 | ✓ |  |  |
| **Cleft Palate and Cleft Lip** | **749** |  |  |  |
| ***Any Cleft Lip/Cleft Palate*** | ***749*** | ✓ |  |  |
| Cleft palate, unilateral, complete | 749.01 | ✓ |  | Cleft Palate |
| Cleft palate, unilateral, incomplete | 749.02 | ✓ |  | Cleft Palate |
| Cleft palate, bilateral, complete | 749.03 | ✓ |  | Cleft Palate |
| Cleft palate, bilateral, incomplete | 749.04 | ✓ |  | Cleft Palate |
| Cleft lip, unilateral, complete | 749.11 | ✓ |  | Cleft Lip or Palate, Cleft Lip |
| Cleft lip, unilateral, incomplete | 749.12 | ✓ |  | Cleft Lip or Palate, Cleft Lip |
| Cleft lip, bilateral, complete | 749.13 | ✓ |  | Cleft Lip or Palate, Cleft Lip |
| Cleft lip, bilateral, incomplete | 749.14 | ✓ |  | Cleft Lip or Palate, Cleft Lip |
| Cleft palate with cleft lip, unilateral, complete | 749.21 | ✓ |  | Cleft Lip or Palate, Cleft Palate |
| Cleft palate with cleft lip, unilateral, incomplete | 749.22 | ✓ |  | Cleft Lip or Palate, Cleft Palate |
| Cleft palate with cleft lip, bilateral, complete | 749.23 | ✓ |  | Cleft Lip or Palate, Cleft Palate |
| Cleft palate with cleft lip, bilateral, incomplete | 749.24 | ✓ |  | Cleft Lip or Palate, Cleft Palate |
| Non-Specific: | 749.00, 749.10, 749.20, 749.25 | ✓ |  |  |
| **Other Congenital Anomalies of Upper Alimentary Tract** | **750** |  |  |  |
| ***Any Gastrointestinal Malformation*** | ***750-751*** | ✓ | ✓ |  |
| Tongue tie | 750.0 |  | ✓ |  |
| Aglossia | 750.11 | ✓ |  |  |
| Congenital adhesions of tongue | 750.12 | ✓ |  |  |
| Fissure of tongue | 750.13 | ✓ |  |  |
| Macroglossia | 750.15 |  | ✓ |  |
| Microglossia | 750.16 |  | ✓ |  |
| Absence of salivary gland | 750.21 | ✓ |  |  |
| Accessory salivary gland | 750.22 | ✓ |  |  |
| Atresia, salivary duct | 750.23 | ✓ |  |  |
| Congenital fistula of salivary gland | 750.24 | ✓ |  |  |
| Congenital fistula of lip | 750.25 | ✓ |  |  |
| Diverticulum of pharynx | 750.27 | ✓ |  |  |
| Tracheoesophageal fistula, esophageal atresia and stenosis | 750.3 | ✓ |  | TE Fistula |
| **Malformation** | **ICD-9-CM Code** | **Major** | **Minor** | **Birth Certificate Designation**  **(if relevant)** |
| Congenital hypertrophic pyloric stenosis | 750.5 |  | ✓ |  |
| Congenital hiatus hernia | 750.6 | ✓ |  |  |
| Non-Specific: | 750.10, 750.19, 750.26, 750.29, 750.4, 750.7, 750.8, 750.9 | ✓ |  |  |
| **Other Congenital Anomalies of Digestive System** | **751** |  |  |  |
| Meckel’s diverticulum | 751.0 |  | ✓ |  |
| Atresia and stenosis of small intestine | 751.1 | ✓ |  | Anal Atresia |
| Anomalies of intestinal fixation | 751.4 | ✓ |  |  |
| Biliary atresia | 751.61 | ✓ |  |  |
| Congenital cystic disease of liver | 751.62 | ✓ |  |  |
| Anomalies of pancreas | 751.7 | ✓ |  |  |
| Non-Specific: | 751.3, 751.5, 751.60, 751.69, 751.8, 751.9 | ✓ |  |  |
| ***Other Gastrointestinal Anomaly*** | ***751*** | ✓ | ✓ | Other Gastrointestinal Anomaly |
| **Congenital Anomalies of Genital Organs** | **752** |  |  |  |
| ***Any Genitourinary Malformation*** | ***752 – 753*** | ✓ | ✓ | Any Genitourinary Malformation |
| ***Malformed Genitalia***  ***1 – Malformed Genitalia***  ***2 – Hypospadia (boys)*** | ***752*** | ✓ | ✓ | Malformed Genitalia |
| Anomalies of ovaries | 752.0 | ✓ |  |  |
| Embryonic cyst fallopian tubes, broad ligaments | 752.11 | ✓ |  |  |
| Doubling of uterus | 752.2 | ✓ |  |  |
| Embryonic cyst of cervix, vagina, and external female genitalia | 752.41 |  | ✓ |  |
| Imperforate hymen | 752.42 |  | ✓ |  |
| Undescended testis | 752.51 |  | ✓ |  |
| Retractile testis | 752.52 | ✓ |  |  |
| Hypospadias | 752.61 | ✓ |  | Infant Hypospadia (boys) |
| Epispadias | 752.62 | ✓ |  |  |
| Congenital chordee | 752.63 |  | ✓ |  |
| Micropenis | 752.64 | ✓ |  |  |
| Hidden penis | 752.65 | ✓ |  |  |
| Indeterminate sex and pseudohermaphroditism | 752.7 | ✓ |  |  |
| Non-Specific: | 752.10, 752.19, 752.3, 752.40, 752.49, 752.69, 752.8, 752.9 | ✓ |  |  |
| **Congenital Anomalies of Urinary System** | **753** |  |  |  |
| Renal agenesis and dysgenesis | 753.0 | ✓ |  | Renal Agenesis |
| Congenital single renal cyst | 753.11 | ✓ |  |  |
| Polycystic kidney, autosomal dominant | 753.13 | ✓ |  |  |
| Polycystic kidney, autosomal recessive | 753.14 | ✓ |  |  |
| Renal dysplasia | 753.15 | ✓ |  |  |
| Medullary cystic kidney | 753.16 | ✓ |  |  |
| Medullary sponge kidney | 753.17 | ✓ |  |  |
| Congenital obstruction ureteropelvic junction | 753.21 | ✓ |  |  |
| Congenital obstruction ureterovesical junction | 753.22 | ✓ |  |  |
| Congenital ureterocele | 753.23 | ✓ |  |  |
| Exstrophy of urinary bladder | 753.5 | ✓ |  |  |
| Atresia and stenosis of urethra, bladder neck | 753.6 | ✓ |  |  |
| Anomalies of urachus | 753.7 | ✓ |  |  |
| Non-Specific: | 753.10, 753.12, 753.19, 753.20, 753.29, 753.3, 753.4, 753.8, 753.9 | ✓ |  |  |
| ***Other Urogenital Anomalies*** | ***753*** | ✓ |  | Other Urogenital Anomalies |
| **Certain Congenital Musculoskeletal Deformities** | **754** |  |  |  |
| ***Any Musculoskeletal Anomalies*** | ***754-756*** | ✓ | ✓ |  |
| ***Any Musculoskeletal Anomalies***  ***(excluding cong. hip displacement)*** | ***754-756***  ***NOT 754.30-754.34*** | ✓ | ✓ |  |
| **Malformation** | **ICD-9-CM Code** | **Major** | **Minor** | **Birth Certificate Designation**  **(if relevant)** |
| ***Musculoskeletal Anomalies***  ***1 – Any***  ***2 – Congenital Hip Displacement***  ***3 – Limb Reduction***  ***4 – Oher Limb Reduction*** | ***754-756*** | ✓ | ✓ | Musculoskeletal Anomalies |
| Of skull, face, and jaw | 754.0 | ✓ |  |  |
| Of sternocleidomastoid muscle | 754.1 | ✓ |  |  |
| Of spine | 754.2 | ✓ |  |  |
| Congenital dislocation of hip, unilateral | 754.30 | ✓ |  |  |
| Congenital dislocation of hip, bilateral | 754.31 | ✓ |  |  |
| Congenital subluxation of hip, unilateral | 754.32 | ✓ |  |  |
| Congenital subluxation of hip, bilateral | 754.33 | ✓ |  |  |
| Congenital dislocation of hip with one subluxation of other hip | 754.34 | ✓ |  |  |
| Genu recurvatum | 754.40 |  | ✓ |  |
| Congenital dislocation of knee (with genu recurvatum) | 754.41 | ✓ |  |  |
| Congenital bowing of femur | 754.42 | ✓ |  |  |
| Congenital bowing of tibia and fibula | 754.43 | ✓ |  |  |
| Talipes varus | 754.50 | ✓ |  |  |
| Talipes equinovarus | 754.51 | ✓ |  | Club Foot |
| Metatarsus primus varus | 754.52 |  | ✓ |  |
| Metatarsus varus | 754.53 |  | ✓ |  |
| Talipes valgus | 754.60 | ✓ |  |  |
| Congenital pes planus | 754.61 | ✓ |  |  |
| Talipes calcaneovalgus | 754.62 | ✓ |  |  |
| Talipes cavus | 754.71 | ✓ |  |  |
| Pectus excavatum | 754.81 |  | ✓ |  |
| Pectus carinatum | 754.82 |  | ✓ |  |
| Non-Specific: | 754.44, 754.59, 754.69, 754.70, 754.79, 754.89 | ✓ |  |  |
| **Other Congenital Anomalies of Limbs** | **755** |  |  |  |
| Polydactyly of fingers | 755.01 | ✓ |  | Polydactyly |
| Polydactyly of toes | 755.02 | ✓ |  | Polydactyly |
| Syndactyly of fingers without fusion of bone | 755.11 | ✓ |  | Syndactyly |
| Syndactyly of fingers with fusion of bone | 755.12 | ✓ |  | Syndactyly |
| Syndactyly of toes without fusion of bone | 755.13 | ✓ |  | Syndactyly |
| Syndactyly of toes with fusion of bone | 755.14 | ✓ |  | Syndactyly |
| ***Limb Reduction*** | ***755.2-755.3***  ***NOT 755.20 or 755.30*** | ✓ |  | Limb Reduction Defect |
| Transverse deficiency of upper limb | 755.21 | ✓ |  | Adactyly |
| Longitudinal deficiency of upper limb, NEC | 755.22 | ✓ |  |  |
| Longitudinal deficiency, combined,involving humerus, radius, and ulna | 755.23 | ✓ |  |  |
| Longitudinal deficiency, humeral, complete or partial | 755.24 | ✓ |  |  |
| Longitudinal deficiency radioulnar, complete or partial | 755.25 | ✓ |  |  |
| Longitudinal deficiency radial, complete or partial | 755.26 | ✓ |  |  |
| Longitudinal deficiency ulnar, complete or partial | 755.27 | ✓ |  |  |
| Longitudinal deficiency carpals or metacarpals, complete or partial | 755.28 | ✓ |  |  |
| Longitudinal deficiency phalanges, complete or partial | 755.29 | ✓ |  |  |
| Transverse deficiency of lower limb | 755.31 | ✓ |  | Adactyly |
| Longitudinal deficiency of lower limb, NEC | 755.32 | ✓ |  |  |
| **Malformation** | **ICD-9-CM Code** | **Major** | **Minor** | **Birth Certificate Designation**  **(if relevant)** |
| Longitudinal deficiency combined, involving femur, tibia, and fibula | 755.33 | ✓ |  |  |
| Longitudinal deficiency femoral, complete or partial | 755.34 | ✓ |  |  |
| Longitudinal deficiency tibiofibular, complete or partial | 755.35 | ✓ |  |  |
| Longitudinal deficiency tibia, complete or partial | 755.36 | ✓ |  |  |
| Longitudinal deficiency fibular, complete or partial | 755.37 | ✓ |  |  |
| Longitudinal deficiency tarsals or metatarsals, complete or partial | 755.38 | ✓ |  |  |
| Longitudinal deficiency, phalanges, complete or partial | 755.39 | ✓ |  |  |
| Congenital deformity of clavicle | 755.51 | ✓ |  |  |
| Congenital elevation of scapula | 755.52 | ✓ |  |  |
| Radioulnar synostosis | 755.53 | ✓ |  |  |
| Madelung’s deformity | 755.54 | ✓ |  |  |
| Acrocephalosyndactyly | 755.55 | ✓ |  |  |
| Accessory carpal bones | 755.56 | ✓ |  |  |
| Macrodactylia (fingers) | 755.57 | ✓ |  |  |
| Cleft hand, congenital | 755.58 | ✓ |  |  |
| Coxa valga, congenital | 755.61 |  | ✓ |  |
| Coxa vara, congenital | 755.62 |  | ✓ |  |
| Congenital deformity of knee (joint) | 755.64 | ✓ |  |  |
| Macrodactylia of toes | 755.65 | ✓ |  |  |
| Anomalies of foot, NEC | 755.67 | ✓ |  |  |
| ***Other Limb Reduction*** | ***755.20, 755.30, 755.4*** | ✓ |  | Other Limb Reduction |
| Non-Specific: | 755.00, 755.10, 755.20, 755.30, 755.4, 755.50, 755.59, 755.60, 755.63, 755.66, 755.69, 755.8, 755.9 | ✓ |  |  |
| **Other Congenital Musculoskeletal Anomalies** | **756** |  |  |  |
| Anomalies of skull and face bones | 756.0 | ✓ |  |  |
| Spondylolysis, lumbosacral region | 756.11 | ✓ |  |  |
| Spondylolisthesis | 756.12 | ✓ |  |  |
| Absence of vertebra, congenital | 756.13 | ✓ |  |  |
| Hemivertebra | 756.14 | ✓ |  |  |
| Fusion of spine (vertebra), congenital | 756.15 | ✓ |  |  |
| Klippel-Feil syndrome | 756.16 | ✓ |  |  |
| Spina bifida occulta | 756.17 |  | ✓ |  |
| Cervical rib | 756.2 |  | ✓ |  |
| Chondrodystrophy | 756.4 | ✓ |  |  |
| Osteogenesis imperfecta | 756.51 | ✓ |  |  |
| Osteopetrosis | 756.52 | ✓ |  |  |
| Osteopoikilosis | 756.53 | ✓ |  |  |
| Polyostotic fibrous dysplasia of bone | 756.54 | ✓ |  |  |
| Chondroectodermal dysplasia | 756.55 | ✓ |  |  |
| Multiple epiphyseal dysplasia | 756.56 | ✓ |  |  |
| Anomalies of diaphragm | 756.6 | ✓ |  | Diaphragmatic Hernia |
| Other congenital anomalies of abdominal wall | 756.79 | ✓ |  | Omphalocele, Gastroschisis |
| Absence of muscle and tendon | 756.81 | ✓ |  |  |
| Accessory muscle | 756.82 | ✓ |  |  |
| Ehlers-Danlos syndrome | 756.83 | ✓ |  |  |
| Non-Specific: | 756.10, 756.19, 756.3, 756.50, 756.59, 756.70, 756.79, 756.89, 756.9 | ✓ |  |  |
|  |  |  |  |  |
| **Malformation** | **ICD-9-CM Code** | **Major** | **Minor** | **Birth Certificate Designation**  **(if relevant)** |
| **Congenital Anomalies of the Integument** | **757** |  |  |  |
| ***Any Skin Anomaly*** | ***757*** | ✓ |  |  |
| Hereditary edema of legs | 757.0 | ✓ |  |  |
| Ichthyosis congenita | 757.1 | ✓ |  |  |
| Dermatoglyphic anomalies | 757.2 | ✓ |  |  |
| Congenital ectodermal dysplasia | 757.31 | ✓ |  |  |
| Vascular hamartomas | 757.32 | ✓ |  |  |
| Congenital pigmentary anomalies of skin | 757.33 | ✓ |  |  |
| Non-Specific: | 757.39, 757.4, 757.5, 757.6, 757.8, 757.9 | ✓ |  |  |
| **Chromosomal Anomalies** | **758** |  |  |  |
| ***Any Chromosomal Anomaly*** | ***758 AND 759.81*** | ✓ |  |  |
| Down’s syndrome | 758.0 | ✓ |  | Down’s Syndrome |
| Patau’s syndrome | 758.1 | ✓ |  |  |
| Edward’s syndrome | 758.2 | ✓ |  |  |
| Autosomal deletion syndromes | 758.3 | ✓ |  |  |
| Balanced autosomal translocation in normal individual | 758.4 | ✓ |  |  |
| Gonadal dysgenesis | 758.6 | ✓ |  |  |
| Klinefelter’s syndrome | 758.7 | ✓ |  |  |
| Fragile X syndrome | 759.83 | ✓ |  |  |
| Non-Specific: | 758.5, 758.81, 758.89, 758.9 | ✓ |  |  |
| ***Other Chromosomal Anomaly*** | ***758*** | ✓ |  | Other Chromosomal Anomaly |
| Prader-Willi Syndrome | 759.81 | ✓ |  |  |
| **Other Congenital Anomalies** | **759** |  |  |  |
| ***Other Congenital Malformations*** | ***759*** | ✓ |  | Other Malformations |
| Anomalies of spleen | 759.0 | ✓ |  |  |
| Anomalies of adrenal gland | 759.1 | ✓ |  |  |
| Situs inversus | 759.3 | ✓ |  |  |
| Conjoined twins | 759.4 | ✓ |  |  |
| Tuberous sclerosis | 759.5 | ✓ |  |  |
| Multiple congenital anomalies, so described | 759.7 | ✓ |  |  |
| Marfan syndrome | 759.82 | ✓ |  |  |
| Non-Specific: | 759.2, 759.6, 759.89, 759.9 | ✓ |  |  |
