# Supplementary material for: Childhood cancer risk in those with chromosomal and non-chromosomal congenital anomalies in Washington State: 1984-2013
Source: PLoS One. 2017 Jun 8;12(6):e0179006. doi: 10.1371/journal.pone.0179006 (PMC5464621; doi:10.1371/journal.pone.0179006)
Supplement: S2 Table — (DOCX) [file pone.0179006.s002.docx]

**S2 Table. Major anomalies among childhood cancer cases diagnosed by ages 5 or 10 years, and their controls, by selected time periods.**

| **Exposure** | **^N cases^** | **^% cases with anomalies^** | **^OR^** | **^(95% CI)^** |
| --- | --- | --- | --- | --- |
| ***Diagnosis age 5 years or younger*** |  |  |  |  |
| Major anomaly | 1952 | 170 (8.7) | 1.63 | 1.39-1.93 |
|  |  |  |  |  |
| Major anomaly *(Restricted to birth years 1989-2009)^a^* | *1584* | *140 (8.8)* | *1.61* | *1.34-1.93* |
|  |  |  |  |  |
| ***Diagnosis age 10 years or younger*** |  |  |  |  |
| Major anomaly | 2583 | 212 (8.2) | 1.54 | 1.33-1.78 |
|  |  |  |  |  |
| Major anomaly *(Restricted to birth years 1994-2004)^b^* | *1250* | *109 (8.72)* | *1.60* | *1.30-1.97* |

*^a^* Excludes children born earlier than 5 years prior to diagnosis, and those without at least 5 years of possible detection in cancer registry.

*^b^* Excludes children born earlier than 10 years prior to diagnosis, and those without at least 10 years of possible detection in cancer registry.
